# A Case of a Thrombotic Storm (Arterial and Venous) in Nephrotic Syndrome

**DOI:** 10.7759/cureus.27505

**Published:** 2022-07-31

**Authors:** Felix Wireko, Sumant Nanduri, Anthony Lyonga Ngonge, Isaac Ikwu, Vishal Poddar

**Affiliations:** 1 Internal Medicine, Howard University Hospital, Washington, D.C., USA; 2 Pulmonary Medicine, Howard University Hospital, Washington, D.C., USA; 3 Pulmonary and Critical Care, Howard University Hospital, Washington, D.C., USA

**Keywords:** primary membranous nephropathy, therapeutic anticoagulation, arterial thromboembolism, venous thromboembolism (vte), nephrotic syndrome

## Abstract

Nephrotic syndrome is a condition characterized by proteinuria, hypoalbuminemia, edema, hyperlipidemia, and a hypercoagulable state. Nephrotic syndrome may lead to several complications, including, but not limited to, increased risk of infection, respiratory distress, and thromboembolism. There are several etiologies of nephrotic syndrome with various predisposing factors ranging from idiopathic, autoimmune diseases, infections (human immunodeficiency virus, hepatitis C virus, hepatitis B virus), drugs, and heavy metal poisoning.

Here, we report the case of a 37-year-old male who presented with worsening exertional dyspnea and bilateral lower extremity swelling. He was found to have simultaneous multiple acute thromboses in both the venous and arterial systems in the setting of worsening renal function. Further investigation revealed that the patient had membranous nephropathy. Initiation of anticoagulation and immunosuppression made a significant difference in his survival.

Vascular thromboembolic (VTE) complications may be the initial presentation that prompts patients with nephrotic syndrome to seek medical care. As such, clinicians must have a high index of suspicion in patients presenting with concurrent VTE and nephrotic-range proteinuria. In addition, given that treatment modalities for the various etiologies of nephrotic syndrome differ considerably, it is also essential to distinguish the type of nephrotic syndrome in a patient, which dictates the treatment algorithm.

## Introduction

Nephrotic syndrome is a clinical syndrome characterized by massive proteinuria (>3.5 g/24 hours), hypoalbuminemia (<30 g/L), hyperlipidemia, edema, and various complications, including, but not limited to, increased risk of infection, respiratory distress, and thromboembolism [1].

Primary nephrotic syndrome is typically caused by minimal change disease, membranous nephropathy, and focal segmental glomerulosclerosis, which is common in children, white, and African American populations, respectively [2].

Thromboembolism is a common and severe complication of nephrotic syndrome, with an incidence of about 25% in the adult population [3]. Urinary anticoagulant loss, especially antithrombin III, elevated fibrinogen levels, and procoagulant protein synthesis, among others, have been suggested as the mechanisms for thromboembolism in nephrotic syndrome [4]. Deep vein thrombosis (DVT), pulmonary embolism (PE), and renal vein thrombosis (RVT) are the common thrombotic complications of nephrotic syndrome, with arterial thrombosis being less prevalent [5]. The femoral artery is the most common site for arterial thrombosis. We present the case of a 37-year-old African American man with biopsy-proven primary membranous nephropathy presenting with simultaneous arterial and venous thromboembolic events.

## Case presentation

A 37-year-old African American man with a history of heart failure with reduced ejection fraction, type 2 diabetes mellitus, hyperlipidemia, and hypertension presented with progressive dyspnea and lower extremity edema for two weeks. A review of the system was negative except for orthopnea. There was no personal or family history of kidney disease.

Emergency room vitals included a significantly elevated blood pressure of 214-250/132-158 mmHg, heart rate of 84 beats per minute, and respiratory rate of 20 breaths per minute. Physical examination was notable for a morbidly obese young man with bilateral dependent pitting edema, as well as dullness to percussion with reduced breath sounds bilaterally in the lung bases.

The initial laboratory investigations were significant for hypokalemia, hyperlipidemia, hypoalbuminemia, elevated serum creatinine, nephrotic-range proteinuria of 5.530 g/g, and elevated B-type natriuretic peptide (Table 1).

**Table 1:** Initial laboratory investigations.

Lab investigations	Day 1	Day 8	Day 15
Sodium	141mEq/L	133 mEq/L	138 mEq/L
Potassium	3.2 mEq/L	3.5 mEq/L	4.5 mEq/L
Chloride	109 mEq/L	97 mEq/L	102 mEq/L
Blood urea nitrogen	46 mg/dL	74 mg/dL	65 mg/dL
Serum creatinine	4.62 mg/dL	5.3 mg/dL	4.12 mg/dL
Calcium	7.5 mg/dL	6.7 mg/dL	7.8 mg/dL
Phosphorus	4.8 mg/dL	6.9 mg/dL	4.4 mg/dL
Brain natriuretic peptide	2,222.2 pg/mL	1,229.1 pg/mL	
Glucose	73 mg/dL	126 mg/dL	159 mg/dL
Troponin	0.11 ng/mL		
White blood cell count	5.43 × 10^9^	10.8 × 10^9^	12.6 × 10^9^
Red blood cell count	3.23 × 10^12^	3.53 × 10^12^	2.66 × 10^12^
Hemoglobin	8.9 g/dL	9.6 g/dL	7.2 g/dL
Hematocrit	27.5%	29.2%	22.3%
Mean corpuscular volume	85.1 fL	82.71fl	83.8fl
Reticulocyte	0.063		
Platelet	257 × 10^9^	171 × 10^9^	110 × 10^9^
Albumin	2.27 g/dL	1.54 g/dL	1.95 g/dL
Total cholesterol	371 mg/dL		
Low-density lipoprotein-cholesterol	249 mg/dL		
High-density lipoprotein-cholesterol	54 mg/dL		
Triglyceride	99 mg/dL		
Vitamin D	<7 ng/mL		
Creatinine phosphokinase	361 IU/L		
Random urine protein	770 mg/dL		
Random urine creatinine	134.63 mg/dL		
Serum C-reactive protein	8.7 mg/dL		
Procalcitonin	9.0 ng/mL	4.47 ng/mL	
Intact parathyroid hormone	59.52 pg/mL		
Cortisol	14.09 µg/dL		
Thyroid-stimulating Hormone	2.34 pg/mL		
Coronavirus disease 2019	Negative		
Pleural fluid analysis			
Appearance	Straw colored		
Albumin	<1.5 g/dL		
Glucose	122 mg/dL		
Total protein	<3 g/dL		
Lactate dehydrogenase	39 IU/L		
White blood cell	67 m^3^		
Red blood cell	74 m^3^		
Serum lactate dehydrogenase	173 IU/L		

**Figure 1:**
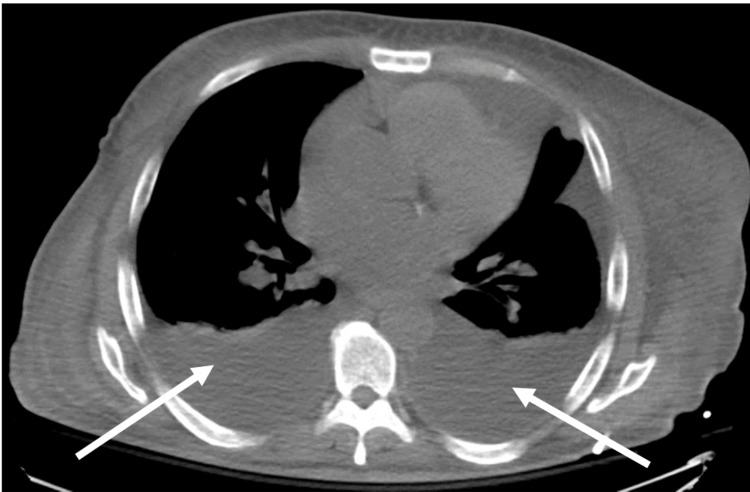
Computed tomography scan of the chest showing bilateral moderate pleural effusion as shown by arrows.

In addition, a chest X-ray showed moderate bilateral pleural effusion, confirmed on computed tomography (CT) scan of the chest (Figure 1).

**Figure 2:**
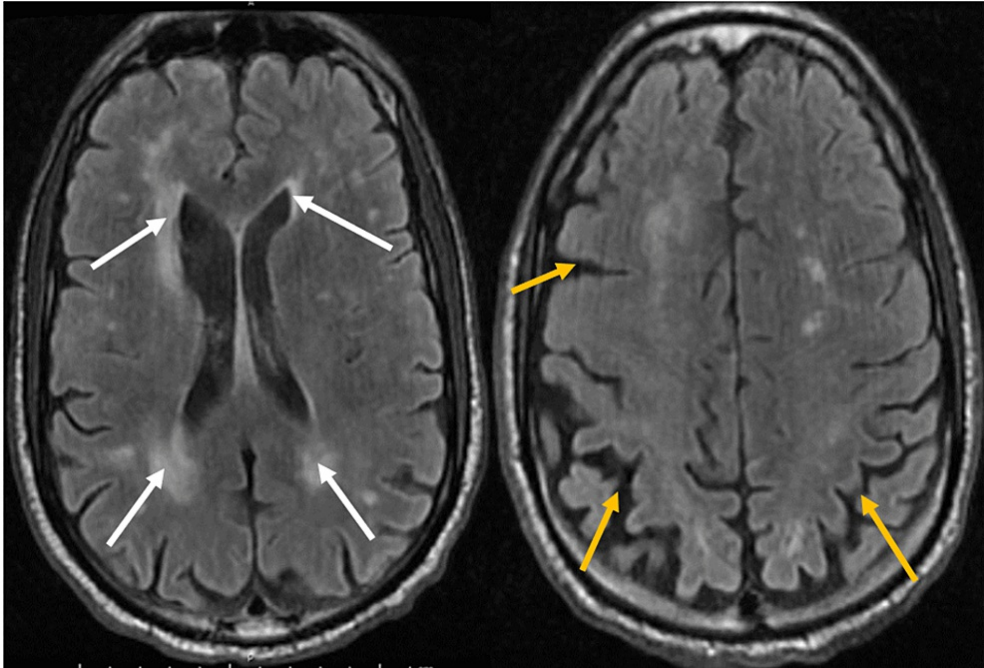
Magnetic resonance imaging of the brain showing multifocal acute bilateral frontal lobe infarcts. Bilateral periventricular and deep white matter, chronic microvascular ischemic changes (white arrows), and generalized cerebral atrophy (yellow).

The patient was admitted for a hypertensive emergency, acute decompensated heart failure, and acute kidney injury (AKI) on stage 2 chronic kidney disease (CKD). Blood pressure was initially controlled on intravenous antihypertensive, and subsequently on oral antihypertensive. The patient had a thoracentesis on the left side. The patient subsequently experienced altered mentation for which a magnetic resonance imaging (MRI) scan of the head showed acute multifocal bilateral frontal infarcts with bilateral periventricular and deep white matter chronic microvascular ischemic changes (Figure 2).

An echocardiogram showed a left ventricular ejection fraction of 25-30%, with a saline contrast study negative for a right-to-left shunt. A transesophageal echocardiogram could not be done because of esophageal ulcers. The patient’s right arm was later noted to be swollen with cephalic vein thrombosis confirmed on a Doppler ultrasound (USG) of the same arm. Subsequent Doppler USG of the lower extremities revealed a non-occlusive DVT of the right common femoral vein. A renal duplex USG scan also revealed a right renal vein thrombosis. The patient was anticoagulated with unfractionated heparin infusion. The patient was worked up for nephrotic syndrome (likely membranous nephropathy) complicated by multiple thromboembolic events. The workup revealed a positive anti-PLA 2R antibody (1:2560) with high specificity for membranous nephropathy. Infectious workup was negative for hepatitis B and C, human immunodeficiency virus, syphilis, and coronavirus disease 2019. Collagen vascular disease workups all returned negative (Table 2).

**Table 2:** Proteinuria workup. AB: antibody; Ig: immunoglobulin; anti-PLA 2R: anti-phospholipase A2 receptor; HIV: human immunodeficiency virus; ANA: antinuclear antibody; anti-GBM: anti-glomerular basement membrane; ANCA: antineutrophil cytoplasmic antibody; RPR: rapid plasma reagin

Laboratory investigation	Results	Reference
Antiphospholipid AB		
Beta 2 glycoprotein I Abs IgA	<2 U/mL	<20 IU/mL
Beta 2 glycoprotein I Abs IgG	<2 U/mL	<20 IU/mL
Beta 2 glycoprotein I Abs IgM	<2 U/mL	<20 IU/mL
Phosphatidylserine IgA	<2 U/mL	<20 IU/mL
Phosphatidylserine IgG	<10 U/mL	<10 IU/mL
Cardiolipin AB IgA	<2 APL-U/mL	<20 APL-U/mL
Cardiolipin AB IgG	<2 GPL-U/mL	<20 GPL-U/mL
Cardiolipin AB IgM	<2 MPL-U/mL	<20 MPL-U/mL
Anti-PLA 2R antibody	Positive titer: (1:2,560)	
Urine electrophoresis		
Albumin: 64%	64%	
Alpha-1 globulin: 1	1%	
Alpha-2 globulin: 5	5%	
Beta globulin: 10	10%	
Gamma globulin: 20	20%	
Protein/Creatinine ratio: 5,530	5,530 mg/g creat	22–120 mg/g creat
Abnormal protein band 1	No M-Spike detected	No M-spike detected
Serum protein electrophoresis		
Ig A	487 mg/dL	47–130 mg/dL
Ig G	1,157 mg/dL	600-1,640 mg/dL
Ig M	45 mg/dL	50–300 mg/dL
Serum immunofixation	No abnormal bands present	No abnormal bands present
Kappa light chains	162 mg/L	3.3–19.4 mg/L
Lambda light chains	131 mg/L	5.7–26.3 mg/L
Complement C3	110.19 mg/dL	79–152 mg/dL
Complement C4	26.77mg/dl	16–38 mg/dL
Ceruloplasmin	20 mg/dL	18–36 g/dL
Copper	35 mg/dL	70-175 mg/dL
Metanephrine	427 pg/mL	36-190 pg/mL
Metanephrine, fractionated plasma	87 pg/mL	<57 pg/mL
Total (MN + NMN)	181 pg/mL	<208 pg/mL
Normetanephrine	94 pg/mL	<148 pg/mL
Hepatitis panel (B and C)	Negative	Negative
HIV	Non-reactive	Non-reactive
ANA	Negative	Negative
Anti dsDNA	Negative	Negative
Anti-GBM	Negative	Negative
Cryoglobulin		
ANCA	Negative	Negative
Myeloperoxidase	<1	<1
Proteinase-3 AB	<1	<1
RPR	Non-reactive	Non-reactive

The patient received doses of methylprednisolone and was started on hemodialysis for worsening renal function and anuria. USG-guided kidney biopsy showed diffusely thickened capillary basement membrane with spikes noted in some loops, interstitium free of significant inflammatory infiltrates, diffuse accumulation of mesangial matrix with evolving nodularity, and extensively effaced podocytes consistent with primary membranous nephropathy. The patient tolerated three times weekly intermittent hemodialysis well with clinical improvement. Heparin drip was subsequently switched to apixaban, and the patient was discharged to a subacute rehabilitation facility.

## Discussion

Though the cause of thromboembolic events in nephrotic syndrome is not fully understood, loss of anticoagulants and increased synthesis of procoagulant precursors are postulated as the likely mechanisms. Membranous nephropathy, hypoalbuminemia (<2 g/dL), and massive proteinuria (>10 g/dL/day) have been identified as independent risk factors for thromboembolic events in nephrotic syndrome [5]. This patient had two of these predictors, namely, membranous nephropathy and hypoalbuminemia.

The risk of thromboembolism in nephrotic syndrome is higher in adults (9%) compared to the pediatric population (1%) and can lead to mortality if left unmanaged [6]. The first month after nephrotic syndrome is critical as most thromboembolic events occur within this period but are the highest within the first six months of diagnosis [7]. In some patients, these complications precede the diagnosis of nephrotic syndrome as they trigger investigations leading to the diagnosis of nephrotic syndrome. This was the case in our patient, where multifocal brain infarcts with venous thromboembolic events (VTEs) coupled with massive proteinuria strengthened our suspicion of nephrotic syndrome and led to its diagnosis. Arterial thromboembolic events (ATEs) in nephrotic syndrome are generally considered rare and less frequent. However, recent evidence suggests a higher incidence per year despite a lower prevalence than VTEs. PE and myocardial infarctions are the most common VTEs and ATEs [8]. However, other studies found RVT as the most common, with varying rates reported in several other studies [9]. The risk of ischemic strokes increases in patients with nephrotic syndrome with age >45 years, male gender, and heart failure [10]. Moreover, proteinuria to serum albumin ratio is a predictor for VTE, while hypertension, diabetes, smoking, estimated glomerular filtration rate, and prior ATE are identified predictors for ATE [8]. Our patient had a combination of these risk factors and developed both VTEs (DVT, RVT) and ATE (ischemic stroke) concurrently. It is, however, not common to have concurrent multiple VTEs and ATEs in the same patient, as was seen in this patient.

While treating the underlying primary membranous nephropathy, anticoagulation is the mainstay treatment for the nephrotic syndrome-associated thromboembolic events. Unfractionated or low-molecular-weight heparin is at the heart of management. Most patients transition to warfarin when stable. However, despite limited evidence for direct-acting oral anticoagulants, its use has been successful in some case reports with good outcomes [9], paving the way for its application clinically in this population.

## Conclusions

Thromboembolic events are common complications of nephrotic syndrome and may be the initial presentation. In addition, the imbalance between anticoagulants and procoagulants is a common trigger for both VTEs and ATEs, especially in the membranous nephropathy subtypes. Therefore, patients presenting with multiple thromboembolic events and proteinuric acute kidney injury should prompt the investigations for nephrotic syndrome as a possible cause.

## References

[1] Tapia C, Bashir K (2022). Nephrotic syndrome. https://www.ncbi.nlm.nih.gov/books/NBK470444/.

[2] McCloskey O, Maxwell AP (2017). Diagnosis and management of nephrotic syndrome. Practitioner.

[3] Kerlin BA, Ayoob R, Smoyer WE (2012). Epidemiology and pathophysiology of nephrotic syndrome-associated thromboembolic disease. Clin J Am Soc Nephrol.

[4] Nakayama T, Mitsuno R, Torimitsu T (2021). Difficulty in managing nephrotic syndrome-associated cerebral venous thrombosis. CEN Case Rep.

[5] Al-Azzawi HF, Obi OC, Safi J, Song M (2016). Nephrotic syndrome-induced thromboembolism in adults. Int J Crit Illn Inj Sci.

[6] Leslom AN, Alrawiah ZM, Al-Asmari AM, Alqashaneen MD, Alahmari AO, Al-Ahmari HO (2020). Prevalence of pulmonary thromboembolism in nephrotic syndrome patients: a systematic review and meta-analysis. J Family Med Prim Care.

[7] Kelddal S, Nykjær KM, Gregersen JW, Birn H (2019). Prophylactic anticoagulation in nephrotic syndrome prevents thromboembolic complications. BMC Nephrol.

[8] Mahmoodi BK, ten Kate MK, Waanders F (2008). High absolute risks and predictors of venous and arterial thromboembolic events in patients with nephrotic syndrome: results from a large retrospective cohort study. Circulation.

[9] Sharp W, Olivero JJ (2018). Venous thrombosis in nephrotic syndrome. Methodist Debakey Cardiovasc J.

[10] Huang JA, Lin CH, Chang YT, Lee CT, Wu MJ (2019). Nephrotic syndrome is associated with increased risk of ischemic stroke. J Stroke Cerebrovasc Dis.

